# Comprehensive Observational Study in a Large Cohort of Asthma Patients after Adding LAMA to ICS/LABA

**DOI:** 10.3390/ph16111609

**Published:** 2023-11-14

**Authors:** Vicente Plaza, Javier Domínguez-Ortega, Diego González-Segura Alsina, Daniele Lo Re, Antoni Sicras-Mainar

**Affiliations:** 1 Servicio de Neumología y Alergia, Hospital de la Santa Creu i Sant Pau, 08025 Barcelona, Spain; vplaza@santpau.cat; 2 Department of Allergy, La Paz University Hospital, Institute for Health Research (IdiPAZ), 28046 Madrid, Spain; javier.dominguez@idipaz.es; 3 Chiesi España, 08908 Barcelona, Spain; d.gonzalez@chiesi.com; 4 Department of Medicinal and Organic Chemistry, Faculty of Pharmacy, Universidad de Granada, 18071 Granada, Spain; lore.daniele.mail@gmail.com; 5 Atrys Health, SA, 28002 Madrid, Spain

**Keywords:** dual therapy, triple therapy, asthma, long-acting muscarinic antagonists, exacerbations, costs

## Abstract

Introduction: Adding LAMA to LABA/ICS is recommended to improve control in patients with persistent asthma. Methods: This observational, retrospective, before-and-after study considered patients diagnosed with asthma who started LABA/ICS + LAMA treatment (triple therapy, TT) between 1 January 2017 and 31 December 2018 and had been treated with LABA/ICS (dual therapy, DT) in the year before. Changes in lung function and exacerbation rates, healthcare resource utilization, and healthcare and non-healthcare costs (€2019) were estimated in patients with asthma in clinical practices in Spain. Data from computerized medical records from seven Spanish regions were collected ±1 year of LAMA addition. Results: 4740 patients (64.1 years old [*SD*: 16.3]) were included. TT reduced the incidence of exacerbations by 16.7% (*p* < 0.044) and the number of patients with exacerbations by 8.5% (*p* < 0.001) compared to previous DT. The rate of patients with severe exacerbations requiring systemic corticosteroids and their hospitalization rates significantly decreased by 22.5% and 29.5%. TT significantly improved FEV_1_, FVC, and FEV_1_/FVC, saving €571/patient for society. Younger patients with asthma (18–44 years old) and patients with severe asthma (FEV_1_ < 60%) performed better upon the initiation of TT. Conclusions: TT reduced asthma exacerbations, improved lung function and reduced healthcare costs vs. DT, particularly in patients requiring systemic corticosteroids to treat severe exacerbations.

## 1. Introduction

Asthma is a heterogeneous disease, usually characterized by chronic airway inflammation. It is defined by a history of respiratory symptoms such as wheeze, shortness of breath, chest tightness and cough, that change in intensity over time, and a varying airflow limitation [1]. Genetic and epigenetic variations, sometimes related to ethnicity, play an important role in asthma susceptibility [2,3]. While children tend to have more genetically driven asthma onset, adults are prone to genetic, environment and comorbidity components interfering with its onset [2]. Epigenetic modifications (DNA hypo- and hypermethylation, histone acetylation/deacetylation/methylation, or variations in microRNA expression) have been observed in epithelial and immune cells [2]. Although asthma prevalence has decreased since 1990, it is the second most prevalent chronic respiratory disease worldwide [4]. It is estimated that 33% of the population between 5–80 years old will develop asthma throughout life, most of them before they reach 20 years old [5]. In Spain, the prevalence of asthma ranges between 2.5% and 13.5%, depending on age, setting, or recording method [6,7]. Despite mortality rates having remained stable for the last 25 years, asthma still causes 2.44 deaths per 100,000 inhabitants, mostly among women [8,9]. According to the Global Burden of Disease Study in 2017, 69% of new cases of chronic respiratory diseases (42,780,000) accounted for asthma; the age-standardized years of life lost rate per 100,000 was 152.8 (108.3 to 195.8) in 2017; and the all-age death rate reached 6.3 (4.3 to 8.2) [10]. Due to its mortality and impact on patients’ quality of life, asthma implies a high burden for healthcare systems and society [4,11,12,13,14]. The clinical burden, which affects even mild asthma patients [15], includes restrictions on daily physical/social activities or sports, and sleep problems [15,16]. Additionally, asthma may result in exacerbations or persistent airflow limitations, greater exposure to oral corticosteroids, increased visits to the emergency department (ED), and hospitalizations [15,16,17].

Asthma is often found to coexist with pulmonary (allergic rhinitis, chronic rhinosinusitis, chronic obstructive pulmonary disease, etc.) or extrapulmonary (gastroesophageal reflux disease, cardiovascular diseases, obesity, etc.) comorbidities [18]. The treatment of some of these comorbidities have managed to decrease exacerbations and airway obstructions, and improve health-related quality of life (HRQoL) and asthma management [18].

The management of asthma aims to achieve good symptom control, and minimize exacerbations, the side effects of treatments, and the risk of mortality [1,7] using pharmacological and non-pharmacological approaches [19]. Treatment with high doses of inhaled corticosteroids (ICS) plus long-acting β2 agonists (LABA) is recommended in severe, persistent asthma. However, in cases where asthma is not well controlled, adding a long-acting muscarinic antagonist (LAMA), like tiotropium or glycopyrronium, to the LABA/ICS therapy, as a single or multiple inhaler triple therapy (MITT), is recommended [1,20,21]. A recent review on uncontrolled asthma patients treated with medium or high doses of ICS + LABA showed that LAMA as an add-on therapy reduced asthma symptoms (assessed by diverse questionnaires), exacerbations, and increased lung function (measured using forced expiratory volume in the first second [FEV_1_], peak expiratory flow and impulse oscillometry parameters, and multiple pulmonary function tests) [22]. LAMA block acetylcholine receptors on airway smooth muscle cells, glands, and nerves, easing muscle contraction and mucus secretion [23].

Treatment with LAMA in combination with LABA/ICS improves lung function and increases the time to severe exacerbations requiring oral corticosteroids [1,20,24]. Furthermore, a recent metanalysis of randomized clinical trials showed that the triple therapy (TT) (LABA/ICS/LAMA) was associated with a reduction in severe exacerbation risk and an improvement in asthma control in comparison to dual therapy (DT) (LABA/ICS) [25]. Another metanalysis showed that triple therapies with medium or high doses of ICS with LABA and LAMA in a single inhaler were effective in achieving asthma control, with no safety concerns [26]. However, the effectiveness of the TT vs. DT in clinical practice has scarcely been analyzed. Suzuki et al. conducted a retrospective, observational study to investigate the treatment patterns and disease burden in patients with asthma who initiated MITT therapy (LABA/ICS + LAMA). They showed that the add-on therapy treatment with tiotropium reduced the incidence of overall exacerbations and exacerbations requiring hospitalization in patients who were being treated with DT [27]. Another study [28] compared the effectiveness and use of healthcare resources when adding tiotropium to ICS/LABA vs. increasing the ICS/LABA dose in a real-world cohort of patients with asthma. The TT had better effectiveness, which implied a reduction in the use of healthcare resources such as ED visits and hospitalizations [28]. Therefore, as add-on therapies, LAMA may reduce the direct and indirect costs associated with the management of asthma. Other randomized controlled studies, gathered in a recent review by Muiser et al. (TALC [*n* = 210], PrimoTinA [*n* = 912], MezzoTinA [*n* = 2103], TRIMARAN/ TRIGGER [*n* = 1155 and 1437, respectively], IRIDIUM [*n* = 3092], ARGON [*n* = 1426], and CAPTAIN [*n* = 2439]) showed that adding LAMA increased lung function, decreased exacerbations, and, in some cases, ameliorated asthma control [29]. Nevertheless, these results have not been confirmed in clinical practice in a large population of patients. Consequently, the aim of this study is to analyze the clinical outcomes, use of healthcare resources, and the costs associated with the addition of LAMA to the LABA/ICS therapy in a wide cohort of patients with asthma (*n* = 4740 patients) in clinical practices in Spain.

## 2. Results

### 2.1. Study Population

**Figure 1:**
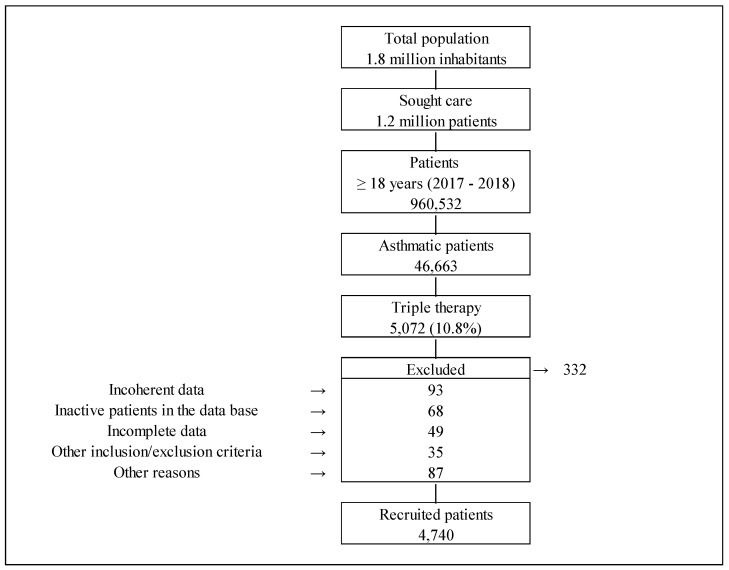
Study scheme.

Out of the 46,663 patients with asthma ≥18 years recorded in the database, 5072 patients (10.8%) were being treated with the TT. According to the inclusion/exclusion criteria, a population of 4740 patients was finally considered in the study (Figure 1).

The study population had a mean age of 64.1 years (*SD*: 16.3), and 63.8% were women. The average time from asthma diagnosis was 32.4 years (*SD*: 15.8). Patients had an average of 2.9 medical conditions (*SD*: 2.0), the most frequent being arterial hypertension (52.3%), dyslipidemia (41.9%) and obesity (24.0%). Other prevalent comorbidities were allergic rhinitis (40.2%) and atopic dermatitis (28.2%). The average Charlson index score was 0.8 (*SD*: 1.4) (Table 1).

### 2.2. Treatments

Before the index date, patients were being treated with a DT, mainly a combination of fluticasone/salmeterol (32.5%), beclomethasone/formoterol (32.0%) or budesonide/formoterol (24.2%). When patients initiated the TT, most kept their DT but there was a slight increase in the use of beclomethasone/formoterol (2.7%) and decreases in the use of fluticasone/salmeterol (3.3%) and budesonide/formoterol (1.2%). The most common LAMA added to the DT was tiotropium (64.0%), followed by glycopyrronium (15.3%) and aclidinium (14.0%) (Table 2). The two last LAMAs were off-label at the time of the study.

Regarding the concomitant treatment, most patients combined the DT with SABA (92.5%), oral corticosteroids (32.4%) and leukotriene receptor antagonists (LRA) (27.7%). When they started the TT, there was a decrease in the use of concomitant medications (*p* ≤ 0.005), except for xanthines. The highest reductions were 12.4% (SABA), 6.3% (systemic antibiotics) and 4.7% (oral corticosteroids) (Table 2).

The median duration of the treatment was 365 days, and the treatment persistence was 56.2% (95% CI: 54.8–57.6%) at 12 months of follow-up (Table S2).

### 2.3. Clinical Outcomes and Deaths

#### 2.3.1. Severe Exacerbations and Deaths

**Figure 2:**
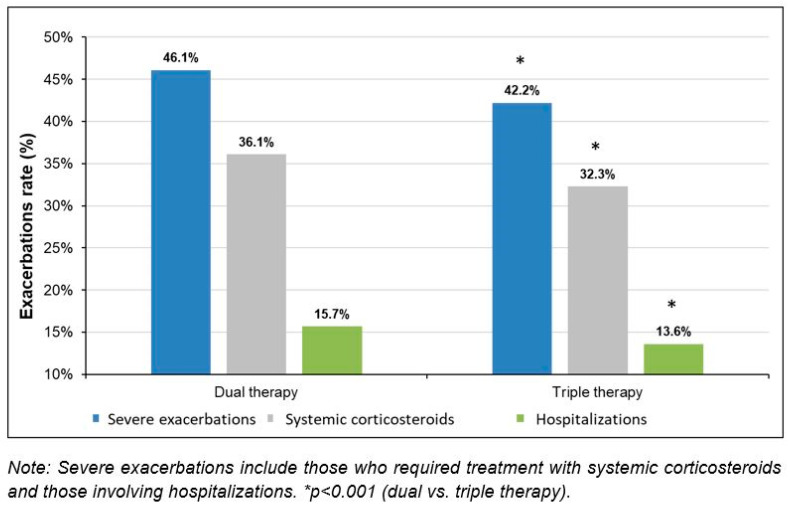
Exacerbation rate according to the two study periods.

During the one-year period with the DT, 46.1% of patients suffered severe exacerbations and the average was 0.6 (*SD*: 0.7) in the study population. However, when they started the TT, the percentage of patients with severe exacerbations was reduced by 8.5% (*p* < 0.001) and the number of severe exacerbations decreased by 16.7% (*p* < 0.044) (Table 3, Figure 2). Severe exacerbations were reduced regardless of the additional diagnosis of COPD (with COPD: 8.6%; *p* < 0.001 and without COPD: 8.5%; *p* < 0.001).

The TT implied a deeper decrease in severe exacerbations in patients with asthma with FEV_1_ at the index date < 60% (12.7%; *p* < 0.001) compared to those with milder asthma (8.9%; *p* < 0.001). In addition, the reductions in severe exacerbation rates varied from 6.0% (≥75 years) to 18.9% (18–44 years; *p* < 0.001 for all comparisons) (Table 4 and Table S3). Our results also showed that patients with asthma with higher Charlson index scores had a predisposition to suffer more severe exacerbations (*p* < 0.001) (Table S3).

Regarding the type of exacerbations, a 22.5% decrease in the incidence rate of patients who required systemic corticosteroids for the treatment of severe exacerbations (*p* < 0.001) and a reduction of 29.5% in the hospitalization rate due to severe exacerbations (*p* < 0.001) (Table 3, Figure 2) were recorded.

During the study period, 3.9% (95% CI: 3.4–4.4%) of the patients died, and the median time to death was 213 days (Table S2).

#### 2.3.2. Lung Function and Eosinophil Counts

In comparison to the DT, the TT with LAMA improved FEV_1_ by 4.3% (*p* < 0.001) and FVC by 1.0% (*p* < 0.001), while FEV_1_/FVC decreased by 0.3% (*p* < 0.004) (Table 3). It was observed that FEV_1_ values increased regardless of the additional COPD diagnosis (with COPD: 4.7%; *p* = 0.003 and without COPD: 4.3%; *p* = 0.008). In addition, a higher increase in FEV_1_ was observed, particularly in patients with more severe asthma (FEV_1_ at index date < 60%) compared to those with FEV_1_ at the index date ≥ 60%. The improvement in FEV_1_ per age group significantly varied between 3.5% (≥75 years) and 8.0% (18–44 years) (Table 4).

The TT also decreased eosinophil counts by 1.5% in comparison to the DT (*p* = 0.046). However, no changes were found in the proportion of patients with eosinophilia (eosinophil counts ≥ 300 cells/μL) (*p* = 0.153) (Table 3).

### 2.4. Use of Healthcare Resources and Costs

The most-required healthcare resources in asthma patients treated with DT were primary care visits and hospitalizations. In general, the TT decreased the use of healthcare resources, particularly the number of patients admitted to hospitals (2.1%; 95% CI: 1.3–3.9%; *p* < 0.001) and the length of hospital stays (1.1 days; 95% CI: 0.7–1.5; *p* < 0.001). The TT also reduced the number of patients on sick leave and the number of sick leave days, although these differences were not statistically significant (*p* = 0.728 and *p* = 0.129, respectively) (Table 5).

Healthcare costs were the highest proportion of total costs associated with the management of the study population. The addition of LAMA decreased healthcare costs by €539 (*p* < 0.001). Total costs amounted to €4719 for patients on DT and €4148 when they were receiving the TT. As a result, the TT implied cost savings of €571 in the management of asthma patients (Table 5).

Cost savings were similar in patients with, or without, a COPD diagnosis. However, these varied from €397 (≥75 years) to €717 (18–44 years; *p* < 0.001 for all comparisons), per age group. In addition, higher reductions in management costs were seen in patients with FEV_1_ at the index date < 60% (€751), compared to patients with FEV_1_ at the index date ≥ 60% (€430; *p* < 0.001 for both comparisons) (Table 4).

## 3. Discussion

The main result of the present study was that the TT reduced the incidence of severe exacerbations by 16.7% (*p* < 0.044) in patients with asthma, and the number of patients who suffered these events decreased by 8.5% (*p* < 0.001). In addition, the MITT registered a higher decrease in severe exacerbations in patients with more severe asthma (FEV_1_ at index date < 60%: 12.7%; *p* < 0.001) compared to those with milder symptoms (8.9%; *p* < 0.001). Therefore, our results confirmed those observed in the phase 3 studies (TRIMARAN and TRIGGER) [20,21], which demonstrated that the TT reduced the rate of severe exacerbations by 23.0% in comparison to the DT (*p* = 0.0076) [20]. In addition, considering that this is a retrospective database study, which is much less restrictive with regard to patient populations compared to the classical Phase 3 trials, better clinical outcomes were registered in patients with a worse health status, such as those with persistent airflow limitations (33.5%; *p* < 0.001) [21]. 

Previous studies also analyzed the effectiveness of LAMA in combination with LABA/ICS for patients with asthma in other countries [27,28,30,31]. Price et al. carried out an observational study in the UK to determine if the addition of LAMA (tiotropium) improved asthma control in routine clinical practice. They included 2042 patients in the study; 67% of these were being treated with ICS/LABA. Their results showed that tiotropium as an add-on therapy decreased the exacerbation rate (relative difference: 27.7%; *p* < 0.001), and prescriptions of oral corticosteroids (absolute difference: 10.3%; *p* < 0.001) and antibiotics (absolute difference: 9.9%; *p* < 0.001) [30]. In comparison to our results, 64% of patients were being treated with tiotropium, and we concluded that the TT reduced the exacerbation rate and the use of concomitant medications such as oral corticosteroids (4.7%; *p* < 0.001) and systemic antibiotics (6.3%; *p* < 0.001). Our results also showed an improvement in lung function (FEV_1_ [*p* < 0.001], FVC [*p* < 0.001], and FEV_1_/FVC values [*p* < 0.004]); however, Price et al. did not find differences in the FEV_1_ values (*p* = 0.935) and FEV_1_/FVC ratio (*p* = 0.382), possibly due to the sample size [30]. 

Asthma implies a high economic burden for the Spanish National Health System and society [12,13,14]. Sicras-Mainar et al. estimated that the management costs of patients with severe asthma amounted to an average of €5493/patient, and that the costs were higher for patients with eosinophilia (€6403/patient; *p* < 0.007) [12]. Another observational study carried out by Melero et al. showed that the annual mean cost of severe asthma was €8554/patient [13]. Our results indicated that the management of patients with asthma treated with DT implied a cost of €4719/patient, but the addition of LAMA resulted in cost savings of €571, which could be even higher in patients with more severe asthma (FEV_1_ < 60%) upon initiation of the treatment (€751/patient). 

To our knowledge, this is the first study to analyze the effectiveness of LAMA as an add-on therapy to ICS/LABA in Spain. This suggests that the next steps should be the use of specific tests to measure whether the observed improvements in asthmatic patients’ clinical outcomes are accompanied by an ameliorated HRQoL in asthmatic patients. There is a wide range of tests to measure treatment impact on patients’ asthma or rhinology control, lung function or HRQoL (asthma control test [ACT], asthma control questionnaire [ACQ], asthma quality of life questionnaire [AQLQ], 22-item sinonasal outcome test [SNOT-22], etc.) [32,33,34]. In addition, a sub-analysis on populations with asthma and specific comorbidities, such as CRS or allergies, could be informative [35]. Patient outcomes could also be determined by the genetic mutations or epigenetic modifications that patients bear and that are involved in asthma mechanisms, predispositions, or even in their relationship to other disorders [35,36,37,38]. The main limitation of this before-and-after study is the lack of a control group towards which we could directly compare results at the time of obtaining the data. Our study was based on registries of a wide population of patients with asthma and described clinical practices in our country, including all LAMA currently prescribed. We considered the same cohort of patients who received both the dual and TT therapies, so they constituted their own control with which to compare the clinical outcomes in both therapies, but the time difference and the individual characteristics of each patient, which may have influenced their development, should be acknowledged. Additionally, our study has the limitations of retrospective and observational studies, such as under-recording of the disorders or clinical outcomes in the database, and variations in physicians’ clinical practices, methods of measurement, and classification/selection biases. Other limitations include the inaccuracy of the disease coding system and the lack of some variables that could influence the results, such as the socioeconomic status of patients.

## 4. Materials and Methods

This is an observational and retrospective study, based on electronic medical records (EMR) from the BIG-PAC^®^ database. The primary data were obtained from the computerized medical records of primary care centers and hospitals (publicly owned services), from integrated health areas in seven Spanish autonomous communities (around 1.8 million patients) [39,40]. These data are anonymized in the source centers in compliance with data protection regulations prior to inclusion in the BIG-PAC^®^ database. Internal studies have shown that information gathered in the BIG-PAC^®^ database is representative of the Spanish population [41].

### 4.1. Study Population and Design

This before-and-after study considered patients with asthma, according to the International Classification of Diseases (9th edition)—Clinical Modification (ICD-9-CM: 493.x), who started a treatment with LABA/ICS + LAMA in a MITT between 1 January 2017 and 31 December 2018 (recruitment period), and who had been previously treated with a single inhaler containing a DT LABA/ICS in the year prior to the index date. We used a before-and-after study design to follow the same individuals, who had uncontrolled asthma, and measure their response after adding LAMA. It should be noted that ICD-9-CM codes 493.x discriminates asthma from other respiratory diseases. The index date was the date when LAMA was added to the treatment. Data of the clinical variables were collected during the year before, and after, the corresponding index date. 

The inclusion criteria were (a) age ≥ 18 years, (b) diagnosis of asthma, (c) being on treatment with LABA/ICS + LAMA (two inhalers) and previous treatment with LABA/ICS (single inhaler), (d) being active in the database for ≥12 months before study initiation, (e) inclusion in the chronic prescription program (with ≥2 prescriptions during the follow-up period) and (f) patients regularly monitored (≥2 records in the database).

The exclusion criteria were: (a) patients on treatment with LABA/ICS + LAMA (one or three inhalers), (b) patients transferred to other centers, displaced or out-of-area, and (c) residents of nursing homes.

### 4.2. Demographic Variables, Comorbidities, and Treatments

Demographic variables (age, gender) and comorbidities (arterial hypertension, dyslipidemia, obesity, diabetes, renal failure, depressive syndrome, heart failure, ischemic heart disease, COPD, peripheral arterial disease, stroke, malignant neoplasms, allergic rhinitis, atopic dermatitis, chronic rhinosinusitis with nasal polyposis, aspirin-exacerbated respiratory disease) were collected. The Charlson comorbidity index was used as a summary variable of comorbidities, as it estimates the mortality risk in connection with comorbidities [42]. Median (P25–P75) time from diagnosis, median BMI, and the status of active smoking were taken.

Treatments (prescribed according to clinical practice) were collected from drug-dispensing records. Drugs were coded using the Anatomical Therapeutic Chemical Classification System (ATC) [43]. Maintenance treatment (ICS/LABA and LAMA [beclomethasone/formoterol, budesonide/formoterol, fluticasone/formoterol, fluticasone/vilanterol, fluticasone/salmeterol, tiotropium, aclidinium, glycopyrronium, umeclidinium] and concomitant medication (oral corticosteroids, chronic use of oral corticosteroids (>6 months), systemic antibiotics, short-acting beta agonists (SABA), short-acting muscarinic antagonists (SAMA), xanthines, LRA, and biologic drugs were considered. 

Treatment persistence/duration was estimated from the index date up to 12 months, or up to the switch to another treatment other than that which motivated inclusion (in the succeeding 30 days), or interruption/discontinuation of medication (≥60 days without renewing the medication) or death, whichever occurred first. Treatment persistence was assessed at 6 and 12 months after the index date. 

### 4.3. Clinical Outcomes and Deaths

Severe exacerbations were defined as a worsening of asthma requiring treatment with systemic corticosteroids (intravenous or oral) for at least three days (with an associated visit to an emergency department or other level of care, or a documented hospital admission). Therefore, two subgroups of severe exacerbations were defined: those who required treatment with systemic corticosteroids and those involving hospitalization [44]. The number and percentage of patients with severe exacerbations, mean (*SD*) severe exacerbations and the number and percentage of patients with 0, 1 or ≥2 severe exacerbations were provided for these two groups and for the overall population with severe exacerbations.

The time from the index date up to the first severe exacerbation was also collected. 

Lung function was estimated using the forced expiratory volume in first, second (FEV_1_, mean, [*SD*] and median [P25–P75]), forced vital capacity (FVC, mean, [*SD*] and median [P25–P75]) and FEV_1_/FVC (mean, [*SD*] and median [P25–P75]). Eosinophil counts in cells/μL (mean [*SD*] and median [P25–P75]) in blood were also measured. The number and percentage of patients with ≥300 cells/μL were also provided.

Deaths were recorded and the time from the index date until the patient’s death was estimated.

### 4.4. Resource Use and Costs

Healthcare and non-healthcare resource use and costs were estimated during the follow-up period. Healthcare resources included medical visits (primary care, specialists [cardiology, internal medicine, endocrinology, vascular, neurology, hematology, geriatrics], and emergency room), hospitalizations (percentage of hospitalized patients and hospitalization days), diagnostic/therapeutic tests (conventional laboratory tests, radiology, computed tomography, magnetic nuclear resonance and other diagnostic/therapeutic tests [catheterization, angioplasty, endarterectomy/thrombectomy] Non-healthcare resources comprised the number on patients on sick leave and the days of work lost.

Cost were expressed in 2019 Euros [12]. Unit costs can be seen in Supplementary Table S1. Drug costs (maintenance treatment [ICS/LABA, LAMA] and concomitant medication including oral corticoids, antibiotics, SABA, SAMA, xanthines, leukotrienes, biologic drugs, and home oxygen therapy, were estimated using the retail price per pack at the time of prescription [45]. The number of days of work disability and the mean salary of the Spanish population were considered to estimate productivity losses (non-healthcare costs) [46].

### 4.5. Statistical Analyses

Descriptive univariate statistical analyses were carried out. The baseline characteristics of patients were summarized using descriptive statistics. For continuous variables, the number of patients, means and standard deviations, were reported. Frequency distributions with quantities and percentages were reported for categorical variables using absolute and relative frequencies (*N*, %). On the other hand, quantitative data were described using means and standard deviations (*SD*) in symmetrical distributions and medians and interquartile ranges (IQR, P25–P75; Q1–Q3) for asymmetrical distributions. The 95% confidence intervals (CI) were calculated to estimate population parameters. 

For the bivariate comparative analysis (before–after study for related groups), the techniques of statistical significance for paired groups were used. McNemar’s tests and Student’s t-tests were conducted to compare treatments, severe exacerbation rates, use of healthcare resources, and costs between the study cohorts (before and after the addition of LAMA to the LABA/ICS therapy). A generalized linear model (GLM) repeated measures procedure was also carried out to compare dependent variables (intra-subject), such as the severe exacerbation rates and the use of SABA and oral corticosteroids, before, and after, the addition of LAMA to the LABA/ICS therapy. A complete factorial model (polynomial contrasts) was developed and the criteria to apply it was the Mauchly’s sphericity test with orthonormalized data transformation. In addition, the impact of the covariates’ inter-subjects (age, Charlson index, differences in costs before and after LAMA treatment and the diagnosis of COPD) in severe exacerbation rates were analyzed. 

Subgroup analyses were developed to estimate the changes in FEV_1_, severe exacerbation rates and costs, per age group (18–44 years, 45–64 years, 65–74 years or ≥75 years), asthma severity (patients with FEV_1_ at the index date <60% or ≥60%) and having an additional diagnosis of chronic obstructive pulmonary disease (COPD). 

The treatment persistence/duration was analyzed using a Kaplan–Meier survival analysis (procedure: log-rank test). Data were censored in the absence of the event. 

The SPSSWIN version 25 statistical program was used. Statistical significance was set at *p* < 0.05.

### 4.6. Compliance with Ethics

This study was carried out in line with the Helsinki Declaration. Patient consent was not obtained since Spanish legislation excludes existing data that are aggregated for analysis. Personal data were de-identified as specified in the Spanish Law 15/1999, of 13 December, on Personal Data Protection, and the Organic Law 3/2018, of 5 December, On the Protection of Personal Data and Guarantee of Digital Rights [47,48]. The study was approved by the Research Ethics Committee of the Hospital de Terrassa, Barcelona, Spain.

## 5. Conclusions

In conclusion, our results showed that the addition of LAMA to the ICS/LABA therapy improved the clinical outcomes of patients with asthma, including lung function and severe exacerbation rates, particularly in younger patients and those with more severe asthma. These improvements led to a decrease in the use of concomitant medications and other healthcare resources, along with cost savings for the Spanish National Health System and society. 

## Figures and Tables

**Table 1:** Baseline characteristics of the study population.

	**Study population**
**Number of patients**	4740
**Demographic characteristics**	
Mean (*SD*) age, years	64.1 (16.3)
Ranges (*n*, %)	
18–44 years	681 (14.4%)
45–64 years	1584 (33.4%)
65–69 years	1090 (23.0%)
≥75 years	1385 (29.2%)
Gender (female) (*n*, %)	3025 (63.8%)
**Comorbidities** (*n*, %)	
Arterial hypertension	2480 (52.3%)
Dyslipidemia	1985 (41.9%)
Obesity	1136 (24.0%)
Diabetes	923 (19.5%)
Renal failure	549 (11.6%)
Depressive syndrome	529 (11.2%)
Heart failure	490 (10.3%)
Ischemic heart disease	474 (10.0%)
COPD	363 (7.7%)
Peripheral arterial disease	316 (6.7%)
Stroke	302 (6.4%)
Malignant neoplasms	298 (6.3%)
**Specific comorbidities** (*n*, %)	
Allergic rhinitis	1906 (40.2%)
Atopic dermatitis	1338 (28.2%)
Chronic rhinosinusitis with nasal polyposis	455 (9.6%)
AERD	109 (2.3%)
**Comorbidities (general)**	
Chronic diseases	
Mean (*SD*)	2.9 (2.0)
Median (P25–P75)	3 (1–4)
Charlson index (mean, *SD*)	0.8 (1.4)
0 (*n*, %)	2902 (61.2%)
1 (*n*, %)	826 (17.4%)
2 (*n*, %)	417 (8.8%)
3+ (*n*, %)	595 (13.0%)
**Other variables**	
Time from diagnosis, years	32.4 (15.8)
Median (P25–P75)	33 (20–45)
BMI, (Kg/m^2^)	28.6 (4.4)
Median (P25–P75)	28 (26–31)
Active smoking (*n*, %)	530 (11.2%)

Values expressed as a percentage (*N*, %) or mean (*SD*). AERD: aspirin-exacerbated respiratory disease; BMI: body mass index; COPD: chronic obstructive pulmonary disease; P: percentiles; *SD*: standard deviation.

**Table 2:** Treatments per group of study.

Study Population (*N* = 4740)	Dual Therapy (before LAMA Treatment)	Triple Therapy	Difference	95% CI	*p*
**Maintenance therapy ^†^**					
Beclomethasone/Formoterol	1516 (32.0%)	1646 (34.7%)	2.7%	1.2%–3.8%	0.005
Budesonide/Formoterol	1147 (24.2%)	1091 (23.0%)	−1.2%	−1.9%–(−1.0%)	<0.001
Fluticasone/Formoterol	151 (3.2%)	185 (3.9%)	0.7%	0.2%–(0.9%)	<0.001
Fluticasone/Vilanterol	385 (8.1%)	435 (9.2%)	1.1%	1.1%–(2.4%)	<0.001
Fluticasone/Salmeterol	1541 (32.5%)	1383 (29.2%)	−3.3%	−5.7%–(−1.5%)	0.005
Tiotropium	---	3032 (64.0%)	---		
Aclidinium *	---	664 (14.0%)	---		
Glycopyrronium *	---	723 (15.3%)	---		
Umeclidinium *	---	321 (6.8%)	---		
**Concomitant therapy**					
Oral corticosteroids	1537 (32.4%)	1316 (27.8%)	−4.7%	−6.4%–(−2.9%)	<0.001
Chronic use of oral corticosteroids (>6 months)	413 (8.7%)	340 (7.2%)	−1.5%	−1.9%–(−1.2%)	<0.001
Systemic antibiotics	1201 (25.3%)	903 (19.1%)	−6.3%	−7.8%–(−4.8%)	<0.001
SABA	4385 (92.5%)	3797 (80.1%)	−12.4%	−14.0%–(−10.2%)	<0.001
SAMA	533 (11.2%)	388 (8.2%)	−3.1%	−5.5%–(−1.2%)	<0.001
Xanthines	184 (3.9%)	184 (3.9%)	0.0%	−0.7%–0.7%	0.999
LRA	1314 (27.7%)	1196 (25.2%)	−2.5%	−3.9%–(−1.0%)	0.001
Biologics	109 (2.3%)	86 (1.8%)	−0.5%	−0.8%–(−0.1%)	0.005

^†^ Multiple inhalers (2 devices, ICS/LABA + LAMA); Values expressed as a percentage (*N*, %). *p*-value: statistical significance (tests for paired groups: McNemar’s tests for qualitative variables and Student’s *t*-tests for quantitative variables). CI: confidence intervals; ICS: inhaled corticosteroids; LABA: long-acting β2 agonist; LAMA: long-acting muscarinic antagonists; LRA: leukotriene receptor antagonists; SABA: short-acting beta agonists; SAMA: short-acting muscarinic antagonists. * Off-label use in asthma.

**Table 3:** Lung function, eosinophil counts, and severe exacerbation rates per study period.

Study Population (*N* = 4740)	Dual Therapy (before LAMA Treatment)	Triple Therapy	Absolute Difference	95% CI	Relative Difference	*p*
**Severe exacerbations**						
*N, % patients with severe exacerbations*	2184 (46.1%)	2002 (42.2%)	−3.9%	−6.0–(−2.7%)	−8.5%	<0.001
Severe exacerbations, mean (*SD*)	0.6 (0.7)	0.5 (0.7)	−0.1 (0.7)	−0.2–0.0	−16.7%	0.044
0 (*n*, %)	2556 (53.9%)	2738 (57.8%)	3.9%	2.7–6.0%	7.2%	<0.001
1 (*n*, %)	1711 (36.1%)	1627 (34.3%)	−1.8%	−2.2–(−1.4%)	−5.0%	0.025
2+ (*n*, %)	473 (10.0%)	375 (7.9%)	−2.1%	−2.5–(−1.7%)	−21.0%	0.018
*N, % patients who required systemic corticosteroids due to severe exacerbations*	1711 (36.1%)	1529 (32.3%)	−3.8%	−5.6–(−2.3%)	−10.5%	<0.001
Severe exacerbations requiring systemic corticosteroids, mean (*SD*)	0.4 (0.5)	0.4 (0.5)	0.1 (0.5)	−0.8–0.2	22.5%	<0.001
0 (*n*, %)	3029 (63.9%)	3211 (67.7%)	3.8%	2.3–(5.6%)	5.9%	<0.001
1 (*n*, %)	1687 (35.6%)	1399 (29.5%)	−6.1%	−7.2–(−5.0%)	−17.1%	<0.001
2+ (*n*, %)	24 (0.5%)	130 (2.8%)	2.1%	1.4–2.8	4.2%	<0.001
*N, % patients admitted to the hospital due to severe exacerbations*	745 (15.7%)	643 (13.6%)	−2.1%	−3.9–(−1.3%)	−13.4%	<0.001
Severe exacerbations requiring hospitalizations, mean (*SD*)	0.3 (0.6)	0.2 (0.4)	−0.1 (1.0)	−0.2–0.0	−29.5%	<0.001
0 (*n*, %)	3995 (84.3%)	4097 (86.4%)	2.1%	1.3–3.9%	2.5%	0.005
1 (*n*, %)	480 (10.1%)	544 (11.5%)	1.4%	0.7–2.1%	13.8%	0.014
2+ (*n*, %)	265 (6.6%)	99 (2.1%)	−4.5%	−5.7–(−3.3%)	−67.2%	<0.001
**Lung function**						
FEV_1_ (mean, *SD*)	55.3 (6.6)	57.6 (6.5)	2.4%	2.2–2.6	4.3%	<0.001
Median (P25–P75)	56 (51–61)	58 (53–63)				
FVC (mean, *SD*)	85.5 (11.2)	86.3 (11.2)	0.8%	0.6–1.1	1.0%	<0.001
Median (P25–P75)	86 (78–93)	86 (79–94)				
FEV_1_/FVC (mean, *SD*)	64.9 (3.7)	64.7 (3.7)	−0.2%	−0.4–(−0.1)	−0.3%	0.004
Median (P25–P75)	65 (62–68)	64 (61–68)				
**Eosinophil counts**						
Eosinophiles in blood (cells/μL)						
Mean (*SD*)	449.9 (165.5)	443.2 (164.4)	−6.7 (9.8)	−13.3–(−0.2)	−1.5%	0.046
Median (P25–P75)	450 (313–589)	443 (307–582)				
Eosinophiles in blood, ≥ 300 cells/μL *n*, %	3681 (77.7%)	3623 (76.4%)	−1.2%	−2.9–(−0.5)	−1.7%	0.153
**Time to first severe exacerbation, days**						
Mean (*SD*)	191.7 (99.1)	183.5 (99.1)	−8.2 (36.6)	−14.1–(−1.8)	−4.3%	0.025
Median (P25–P75)	191 (104–278)	184 (97–269)				

Values expressed as a percentage (*N*, %) or mean (*SD*). *p*-value: statistical significance (tests for paired groups: McNemar’s tests for qualitative variables and Student’s *t*-tests for quantitative variables). CI: confidence intervals; diff: difference; FEV1: forced expiratory volume in first second; FVC: forced vital capacity; LAMA: long-acting muscarinic antagonists; P: percentiles; *SD*: standard deviation.

**Table 4:** Subgroup analyses according to diagnosis of COPD, age, and asthma severity.

Study Population (*N* = 4740)	Dual Therapy (before LAMA Treatment)	Triple Therapy	Absolute Difference	95% CI	Relative Difference	*p*
**Diagnosis of COPD**						
*FEV_1_*						
Without COPD	55.3 (6.6)	57.7 (6.5)	2.4	2.3–2.4	4.3%	0.008
With COPD	53.5 (6.5)	56.0 (6.4)	2.5	2.3–2.7	4.7%	0.003
*Severe exacerbations*						
Without COPD	46.2%	42.3%	−3.9%	−5.0–(−2.8%)	−8.5%	<0.001
With COPD	47.5%	43.4%	−4.1%	−5.0–(−3.2%)	−8.6%	<0.001
*Costs*						
Without COPD	€4711	€4145	−€565	−829 €–(−300 €)	−12.0%	<0.001
With COPD	€4959	€4356	−€603	−982 €–(−224 €)	−12.2%	<0.001
**Age**						
*FEV_1_ (mean, SD)*						
18–44 years	57.4 (6.3)	62.0 (6.6)	4.6	4.2–5.0	8.0%	<0.001
45–64 years	56.0 (6.1)	59.9 (6.0)	3.9	3.5–4.3	7.0%	<0.001
65–74 years	55.6 (6.7)	57.9 (6.4)	2.3	1.2–2.4	4.1%	0.009
75+ years	53.8 (6.6)	55.7 (6.4)	1.9	0.8–3.0	3.5%	0.028
*Severe exacerbations*						
18–44 years	41.6%	33.7%	−7.9%	−10.1–(−5.7%)	−18.9%	<0.001
45–64 years	43.6%	35.9%	−7.7%	−9.9–(−5.5%)	−17.7%	<0.001
65–74 years	46.8%	42.7%	−4.1%	−5.4–(−2.8%)	−8.7%	<0.001
75+ years	49.1%	46.2%	−3.0%	−3.6–(−2.4%)	−6.0%	<0.001
*Costs (€)*						
18–44 years	€4019	€3303	−€717	−949 €–(−485 €)	−17.8%	<0.001
45–64 years	€4354	€3686	−€669	−1102 €–(−235 €)	−15.4%	<0.001
65–74 years	€4891	€4400	−€491	−768 €–(−214 €)	−10.0%	<0.001
75+ years	€5387	€4990	−€397	−687 €–(−108 €)	−7.4%	<0.001
**Asthma severity**						
*FEV_1_*						
FEV1 ≥ 60% at index date	62.1 (2.8)	64.5 (3.1)	2.4	1.2–3.6	3.9%	0.022
FEV1 < 60% at index date	50.0 (5.3)	57.2 (5.2)	7.2	6.8–7.6	14.4%	<0.001
*Severe exacerbations*						
FEV1 ≥ 60% at index date	44.0%	40.1%	−3.9%	−4.9–(−2.9%)	−8.9%	<0.001
FEV1 < 60% at index date	48.2%	42.1%	−6.1%	−7.5–(−4.7%)	−12.7%	<0.001
*Costs*						
FEV1 ≥ 60% at index date	€4585	€4155	−€430	−625 €–(−234 €)	−9.4%	<0.001
FEV1 < 60% at index date	€4966	€4214	−€751	−1167 €–(−335 €)	−15.1%	<0.001

CI: confidence intervals; diff: difference; COPD: chronic obstructive pulmonary disease; FEV_1_: forced expiratory volume in first second; LAMA: long-acting muscarinic antagonists.

**Table 5:** Use of healthcare and non-healthcare resources in both study periods.

Study Population (*N* = 4740)	Dual Therapy (before LAMA Treatment)	Triple Therapy	Difference	95% CI	*p*
**Use of healthcare and non-healthcare resources ^†^**					
Primary care medical visits (mean, *SD*)	10.4 (9)	9.9 (10.5)	−0.5 (9.1)	−0.8–(−0.3)	<0.001
Specialized care medical visits (mean, *SD*)	1.2 (3.5)	1 (1.5)	−0.2 (3.4)	−0.3–(−0.2)	<0.001
Emergency medical visits (mean, *SD*)	0.7 (1.6)	0.6 (0.9)	−0.1 (1.7)	−0.2–0.0	0.035
Hospitalized patients (*n*, %)	745 (15.7%)	643 (13.6%)	−2.1%	−3.9–(−1.3%)	<0.001
Hospitalization days (mean, *SD*)	4.6 (12.8)	3.6 (10.6)	−1.1 (15)	−1.5–(−0.7)	<0.001
Laboratory tests (mean, *SD*)	1.3 (1.7)	1.5 (2.0)	0.2 (2.0)	0.1–0.3	<0.001
Conventional radiology (mean, *SD*)	0.6 (0.8)	0.3 (0.6)	−0.2 (1)	−0.3–(−0.2)	<0.001
Computed tomography (mean, *SD*)	0.4 (0.5)	0.3 (0.8)	−0.1 (0.9)	−0.3–(−0.1)	<0.001
Magnetic nuclear resonance (mean, *SD*)	0.2 (0.4)	0.1 (0.3)	−0.1 (0.5)	−0.3–(−0.1)	<0.001
Other diagnostic/therapeutic tests (mean, *SD*)	2.4 (0.5)	2.4 (0.5)	0 (0.4)	−0.1–0.0	0.006
Patients on sick leave (*n*, %)	612 (12.9%)	602 (12.7%)	−0.2%	−1.5–(−0.9)	0.728
Sick leave days (mean, *SD*)	3.1 (10.7)	2.8 (10.8)	−0.3 (14.3)	−0.7–(−0.1)	0.129
**Healthcare and non-healthcare costs (€) ^†^**					
Primary care medical visits (mean, *SD*)	251 (219)	239 (254)	−11.8 (220)	−18.1–(−5.5)	<0.001
Specialized care medical visits (mean, *SD*)	120 (336)	99 (144)	−20.7 (330.8)	−30.1–(−11.3)	<0.001
Emergency medical visit (mean, *SD*)	82 (195)	75 (107)	−6.3 (204.8)	−12.1–(−0.4)	0.035
Hospitalizations (mean, *SD*)	2638 (7298)	2018 (6042)	−620.2 (8533.2)	−863.2–(−377.3)	<0.001
Laboratory tests (mean, *SD*)	44 (58)	50 (68)	6.1 (67.9)	4.1–8.0	<0.001
Conventional radiology (mean, *SD*)	17 (24)	10 (18)	−7.3 (28.8)	−8.1–(−6.5)	<0.001
Computed tomography (mean, *SD*)	39 (49)	28 (76)	−11.5 (86.4)	−13.9–(−9.0)	<0.001
Magnetic nuclear resonance (mean, *SD*)	38 (75)	22 (60)	−16.1 (90.3)	−18.7–(−13.5)	<0.001
Other diagnostic/therapeutic tests (mean, *SD*)	116 (25)	116 (25)	0.7 (17.9)	0.2–1.2	0.006
Drugs (mean, *SD*)	984 (1787)	1209 (1572)	225.1 (1431.9)	184.3–265.8	<0.001
Healthcare cost (mean, *SD*)	4406 (7870)	3867 (6602)	−538.8 (9002.4)	−795.1–(−282.4)	<0.001
Non-healthcare cost (productivity loss) (mean, *SD*)	313 (1087)	281 (1094)	−32 (1451.5)	−73.4–(−9.3)	0.129
Total cost (mean, *SD*)	4719 (8110)	4148 (6711)	−570.8 (8995.9)	−827.0–(−314.7)	<0.001

^†^ calculated 12-month period after the index date. Values expressed as a percentage (*N*, %) or mean (*SD*). *p*-value: statistical significance (tests for paired groups: McNemar’s tests for qualitative variables and Student’s *t*-tests for quantitative variables). CI: confidence intervals; LAMA: long-acting muscarinic antagonists; *SD*: standard deviation.

## Data Availability

Data are contained within the article and Supplementary Materials.

## Supplementary Materials

The following supporting information can be downloaded at: https://www.mdpi.com/article/10.3390/ph16111609/s1, Table S1: Unit costs (€2019); Table S2: Treatment persistence and deaths during the study period; Table S3: Generalized linear model repeated measures.
