# Supplementary material for: Comprehensive Observational Study in a Large Cohort of Asthma Patients after Adding LAMA to ICS/LABA
Source: Pharmaceuticals (Basel). 2023 Nov 14;16(11):1609. doi: 10.3390/ph16111609 (PMC10675027; doi:10.3390/ph16111609)
Supplement: Supplementary file 1 [file pharmaceuticals-16-01609-s001.zip › pharmaceuticals-2684205-supplementary.pdf]

Comprehensive Observational Study in a Large Cohort of Asthma Patients after Adding LAMA to ICS/LABA

SUPPLEMENTARY MATERIAL

Table S1. Unit costs (€) for healthcare and non-healthcare resources.

Healthcare and non-healthcare resources	Unit costs (€)
**Medical visits**	
Primary care visit	24.2
Emergency visit	122.8
Hospitalizations (per day)	568.0
Specialized care medical visit*	96.7
**Complementary tests**	
Laboratory tests	33.8
Conventional radiology	29.8
Computed tomography	100.3
Magnetic nuclear resonance	185.0
Other diagnostic/therapeutic tests	49.2
**Drugs**	*Retail price*
**Productivity loss (indirect costs)**	
Cost per day of sick leave	101.2

Sources: Sicras-Mainar et al., 2019[40] and National Statistics Institute[46]. *Regarding the Pneumology, Allergology and Internal Medicine Departments.

Table S2. Treatment persistence and deaths during the study period.

	Study population (N = 4740)
**Treatment persistence**	
Duration of treatment, days	
Mean (*SD*)	284.8 (111.2)
Median (P25 - P75)	365 (203 – 365)
Treatment persistence, patients	
6 months	3696 (78.0%)
95% CI	76,8 – 79.2%
12 months	2663 (56.2%)
95% CI	54.8 – 57.6%
Discontinuation after 12 months	2077 (43.8%)
**Deaths**	
Percentage	186 (3.9%)
95% CI	3.4 – 4.4%
Time to death, days	
Mean (*SD*)	203.3 (93.6)
Median (P25 - P75)	213 (127 – 278)

Values expressed as a percentage (*N*, %) or mean (*SD*). CI: confidence intervals; P: percentiles; *SD*: standard deviation.

Table S3. Generalized linear model repeated measures.

**Contrasts intra-subject (dependent variables)**	**SC (Tipo III)**	**F**	***p***
1. Number of severe exacerbations (before/after LAMA treatment)	95.220	571.551	<0.001
2. Use of SABA (before/after LAMA treatment)	9.809	31.857	<0.001
3. Use of oral corticosteroids (before/after LAMA treatment)	5.596	11.643	0.001
**Contrasts inter-groups (covariates)**	**SC (Tipo III)**	**F**	***p***
Age, years	4.334	9.016	0.003
Charlson index	13.512	28.113	<0.001
Difference in costs before/after LAMA treatment, euros	20.696	43.059	<0.001
Diagnosis of COPD	0.303	1.329	0.249

Variables included according to the established entry order. SCs were estimated using a Type III (balanced model with no empty cells). COPD: chronic obstructive pulmonary disease; F: Snedecor's F distribution; LAMA: long-acting muscarinic antagonists; P: statistical significance SABA: short-acting beta agonists; SC: sum of squares.
